# Peripheral Blood Leukocyte Detection Based on an Improved Detection Transformer Algorithm

**DOI:** 10.3390/s23167226

**Published:** 2023-08-17

**Authors:** Mingjing Li, Shu Fang, Xiaoli Wang, Shuang Chen, Lixia Cao, Jinye Han, Haijiao Yun

**Affiliations:** School of Electronic Information Engineering, Changchun University, Changchun 130000, China; limj@ccu.edu.cn (M.L.);

**Keywords:** white blood cell, Fourier ptychographic microscopy, DETR, target detection

## Abstract

The combination of a blood cell analyzer and artificial microscopy to detect white blood cells is used in hospitals. Blood cell analyzers not only have large throughput, but they also cannot detect cell morphology; although artificial microscopy has high accuracy, it is inefficient and prone to missed detections. In view of the above problems, a method based on Fourier ptychographic microscopy (FPM) and deep learning to detect peripheral blood leukocytes is proposed in this paper. Firstly, high-resolution and wide-field microscopic images of human peripheral blood cells are obtained using the FPM system, and the cell image data are enhanced with DCGANs (deep convolution generative adversarial networks) to construct datasets for performance evaluation. Then, an improved DETR (detection transformer) algorithm is proposed to improve the detection accuracy of small white blood cell targets; that is, the residual module Conv Block in the feature extraction part of the DETR network is improved to reduce the problem of information loss caused by downsampling. Finally, CIOU (complete intersection over union) is introduced as the bounding box loss function, which avoids the problem that GIOU (generalized intersection over union) is difficult to optimize when the two boxes are far away and the convergence speed is faster. The experimental results show that the mAP of the improved DETR algorithm in the detection of human peripheral white blood cells is 0.936. In addition, this algorithm is compared with other convolutional neural networks in terms of average accuracy, parameters, and number of inference frames per second, which verifies the feasibility of this method in microscopic medical image detection.

## 1. Introduction

Medical imaging has become an important part of clinical diagnosis and biomedical research, which can help doctors to study, monitor, and diagnose diseases. Peripheral blood usually refers to the human body in addition to bone marrow outside of the blood, which mainly contains platelets, red blood cells, and white blood cells [1]. A routine blood test is one of the three major clinical tests [2]. It is mainly conducted through an analysis of three kinds of cells in peripheral blood to determine whether there is a disease [3]. In the medical field, the analysis of WBCs is of great significance for the diagnosis of disease because they play an important role in the human immune system. If infection occurs, there will be abnormal WBC values [4]. Acute leukemia, aplastic anemia, malignant histiocytosis, and other diseases are caused by leukopenia [5]. Commonly seen in infectious diseases, bacterial and viral infections can cause elevated white blood cells. When the infection is controlled, white blood cells will return to normal levels. Therefore, the detection of WBCs in clinical practice can help doctors make correct diagnoses.

At present, the detection and identification of WBCs mainly rely on the use of blood cell analyzers and artificial microscopy [6]. Artificial microscopy is the ‘gold standard’ of clinical examination, which requires sufficient theory and experienced professionals to operate. The inspection process is time-consuming and laborious, and the results are easily affected by human factors. Mistakes and omissions often occur. A blood cell analyzer is an instrument widely used in hospitals. It has the advantages of high precision and fast speed, but its cost is high, and it cannot evaluate the morphology of WBCs. When abnormal WBCs are detected, artificial microscopy is also needed to assist in testing. It is not only necessary to train experienced doctors but also to buy expensive instruments, which is a huge burden for hospitals.

With the rapid development of image processing technology, the ability to detect WBCs is becoming better and faster. To realize WBC detection, the first step is to collect cell images [7]. In order to obtain a clear microscopic image of WBCs, a 20-fold objective of a common microscope is usually used. However, the field of view of a 20-fold objective lens is too small. In order to detect a sufficient number of white blood cells, the sample needs to be mechanically scanned, and mechanical motion is detrimental to the accuracy of WBC detection. In addition, when collecting images, the thickness of the blood is not uniform, which will cause the image to lose focus in thicker areas of the sample. Therefore, it is necessary to focus on the objective lens, and this repeated image acquisition process is time-consuming and laborious. The above problems show that there are many challenges in the acquisition of white blood cell images using an ordinary microscope.

Here, the introduction of Fourier ptychographic microscopy (FPM) [8], as a solution, can objectively solve the problems of leukocyte image acquisition. FPM is a recently developed imaging technique [9] that can increase the numerical aperture of the microscope system without the need for mechanical scanning to obtain high-resolution, wide-field images synthesized from low-resolution images. FPM is a simple modification of the traditional microscope, which only needs to replace the ordinary light source with a programmable light-emitting diode (LED) array and add a camera. Thanks to minor modifications to conventional microscopes, FPM offers a flexible and low-cost method compared to the more expensive precision mechanical instruments that are usually involved.

Recently, deep learning has significantly improved the level of object detection. According to the different structures of the model, object detection can be divided into two types based on a convolutional neural network (CNN) and a transformer. Among them, the CNN’s success in image recognition mainly lies in its powerful capabilities of bias, activation function, and filling encoded using a convolutional layer [10]. Recently, a transformer was used as a self-attention mechanism to capture global feature information, showing higher performance than the CNN [11]. The single-stage target detection network based on the CNN, such as YOLO [12], directly regresses the target size, location, and category to the candidate box, and the detection speed is fast, but the accuracy is low. Two-stage networks such as the Faster R-CNN [13] need to generate candidate boxes first and then classify and regress each candidate box. The detection accuracy is high, but the speed is slow. The white blood cell detection method based on the CNN is still insufficient; the model is susceptible to factors such as artificially designed steps and post-processing, and its convergence speed and target detection ability need to be improved. Transformer-based DETR [14] is an end-to-end object detection model that regards object detection as a set of prediction problems rather than a traditional bounding box regression problem. It uses the ensemble prediction method to directly provide the prediction results, which saves the manual design stage and post-processing process.

In short, an accurate and efficient calculation method is needed to support peripheral blood leukocyte detection, especially to achieve complete end-to-end automation. In this paper, improved DETR peripheral blood leukocyte detection is combined with the advantages of FPM to realize the detection of peripheral blood leukocytes. The experimental results verify the effectiveness of the algorithm in detecting white blood cells and can assist doctors in diagnosing diseases clinically.

In this paper, the research background, motivation, and purpose are introduced in Section 1. In Section 2, relevant works in the literature are investigated, and their advantages and disadvantages are analyzed. Then, the proposed method and architecture are described in Section 3. The experimental process and results are shown and discussed in Section 4. Finally, the conclusion is drawn in Section 5.

## 2. Related Works

### 2.1. Fourier Ptychographic Microscopy

The traditional optical microscope consists mainly of a camera, objective lens, and lens barrel, with the performance primarily dependent on the objective lens. Generally, the microscope’s performance is evaluated based on two factors: resolution and field of view. Resolution is determined by the numerical aperture of the objective, while the field of view is determined by the aperture of each lens in the objective. From the perspective of a microscopic imaging system, spatial resolution and imaging field of view are a pair of irreconcilable contradictions. In order to observe the tiny details of the sample, it is necessary to increase the numerical aperture of the microscope objective and improve its resolution. However, this imaging field of view will become smaller after such an imaging operation. When observing an object through a low-power lens, the whole picture of the measured object can be observed, but the details are not visible. The high-power lens can clearly see the details of the measured object, but the entire object cannot be viewed simultaneously.

In 2013, Professor Zheng Guoan and his team from the California Institute of Technology proposed a novel computational microscopy method with a wide field of view and high resolution, which is called FPM [8]. This method breaks through the limitations of traditional optical microscopes by using synthetic aperture, optimization theory, and other related technologies to obtain high-resolution images using low-magnification objective lenses. Based on the advantages of FPM, in just a few years of development, it has been applied in many fields, such as bioimaging of living samples [15], cell detection [16], cell counting [17,18], and digital pathology [19]. In recent years, FPM has been improved in terms of implementation methods [20,21,22], imaging performance [23,24,25], and reconstruction efficiency [26,27,28]. These applications and improvements in biomedicine also further reflect the great potential of FPM.

The introduction of FPM can solve the problems that exist in the current acquisition of white blood cell images. It can significantly improve the image resolution of the traditional microscopic system and reduce the complexity of the experimental structure by simply changing the light source structure. FPM has a simple light source structure and equipment price, and it can obtain white blood cell images with high resolution and a wide field of view. By utilizing its advantages and combining them with the excellent performance of deep learning networks in target detection tasks, the detection of white blood cells in peripheral blood is achieved.

### 2.2. Blood Cell Detection

Artificial microscopy not only consumes a significant amount of manpower and material resources, but the test results are also easily affected by human factors. The blood cell analyzer is unable to observe the morphology of white blood cells, and there are still some shortcomings auxiliary in medical diagnosis. With the rapid advancement of digital information and deep learning in the field of computer vision, it has become a major development that uses computer graphics to assist doctors in detecting the quantity and morphology of white blood cells.

The study of red blood cell counting methods began internationally as early as 1852. In 1855, a counting plate specifically dedicated to counting blood cells was introduced [29]. At present, there are two different methods for blood cell detection: one involves detecting blood cells through image processing methods, while the other is based on deep learning methods.

#### 2.2.1. Methods Based on Image Processing

Cuevas et al. proposed an automatic algorithm for detecting white blood cells embedded in complex images. The algorithm utilizes the DE (differential evolution) algorithm to optimize the encoded candidate ellipse set, allowing it to adapt to the white blood cells present in the edge mapping of the smear image [30]. Kasim employs a hybrid spatial learning structure consisting of K-means and maximizing expectations to obtain regions of interest. This method minimizes the quality of dyeing and lighting problems [31]. Cheng proposed a novel type of fuzzy morphological neuron model network. The algorithm converts the image’s RGB (red, green, blue) color space into the HSL (hue, saturation, lightness) color space and achieves white blood cell recognition through fuzzy morphological network pairs [32]. Lin et al. proposed a feature weight adaptive K-means clustering-based algorithm for extracting complex white blood cells. Before extracting white blood cells, color space decomposition and K-means clustering are combined for image segmentation. Then, the complex white blood cells are separated again based on the watershed algorithm and finally classified [33].

Although the above methods can achieve the detection of white blood cells, these methods can only be detected after the completion of cell color space conversion or cell segmentation. Furthermore, the detection effect is easily influenced by the results of image processing. Additionally, this method requires a significant amount of operations, and the process is cumbersome, resulting in low efficiency of cell detection. Therefore, it is unlikely to be applicable to the clinical diagnosis workflow.

#### 2.2.2. Method Based on Deep Learning

The target detection algorithm of traditional convolutional neural network. Namdev et al. proposed a new neural network classifier for bone marrow leukocyte classification. They introduced the FGS (fractional gravitational search) algorithm into the weight update algorithm [34]. Huang et al. proposed a white blood cell classification manifold learning method based on an attention-aware residual network. This method adaptively learns discriminative features between classes through the attention module to classify white blood cells [35]. Yao et al. utilized a two-mode weighted optimal deformable convolutional neural network to classify white blood cells [36]. However, the traditional deep convolution algorithm is limited by the size of the receptive field of the convolution kernel and the shape of the anchor frame and lacks the ability to learn global features. Therefore, it has a low accuracy of target detection.

At present, the target detection algorithms based on deep learning are basically divided into two categories. The first category consists of single-stage target detection algorithms like YOLO [37,38,39] and SSD [40]. The second category includes two-stage detection algorithms like Faster-R-CNN [13], which utilize a candidate region network to extract candidate target information. Liu et al. proposed the use of Faster R-CNN to detect and count red blood cells [41], which has proven to be effective in identifying red blood cells. However, the Faster R-CNN uses candidate regions to extract the information of the target of interest; the detector consumes a significant amount of computing resources and has a low detection rate. At the same time, this method has a high rate of missed detection areas of the image with overlap and density. Zhang et al. proposed a cell counting method that is based on YOLO density estimation [42]. This method modifies the backbone network of YOLO to detect cells. While it can effectively enhance the target detection rate, the overall network level of the YOLO series is simplistic, and the backbone network lacks insufficient feature extraction capability. In addition, the combination of convolution and upsampling in the neck fails to effectively integrate high-quality context feature information, leading to low overall detection accuracy.

The object-detection method based on the transformer network structure has gained significant attention and research due to the continuous development of deep learning. The transformer utilizes the attention mechanism to acquire image features and adjusts the weight parameters through the dot operation to reduce the learning bias of the model. Therefore, the transformer has a more powerful generalization ability compared to the CNN. Sun et al. introduced a blood cell image recognition method that builds upon the improved vision transformer [43]. They incorporated a sparse attention module into the self-attention mechanism transformer module. The model’s performance was evaluated using the Munich blood cell morphology dataset. Although this method demonstrates superior performance compared to the CNN, it requires improvement in terms of convergence speed and small target detection ability due to the impact of network depth and model parameters. The target detection model is susceptible to missing the target due to significant variations between different cell instances. Furthermore, there exists an imbalance between the positive and negative samples of the target instance and the background region in the cell image leading to a decrease in detection accuracy. Accuracy and robustness still exhibit noticeable deficiencies in the detection of white blood cells.

### 2.3. Summary

In order to effectively address the limitations of existing research, this paper improves detection efficiency from two aspects. Firstly, it utilizes the FPM system to collect cell images. Secondly, it enhances the target detection model DETR. This method has several advantages for the research object, which is the detection of peripheral blood leukocytes. The introduction of FPM solves the issue of incompatibility between the field of view and resolution in leukocyte image acquisition. It enables the acquisition of white blood cell images with a wide field of view and high resolution. This method does not require any color conversion or binary segmentation, and the entire process is fully automated, fast, and accurate. The DETR algorithm does not rely on artificially designed anchor frames. This reduces the requirement for prior knowledge of peripheral blood leukocyte samples and improves the model’s ability to generalize.

The main improvement strategies are as follows:

1. In the ResNet50 network structure, the convolution of the Conv Block residual structure has been improved, and the average pooling operation is used at the residual edge. This helps reduce the problem of losing small target feature information during downsampling and effectively reduces overfitting, thereby improving target detection performance;
2. The GIOU loss function has been replaced with CIOU to address the difficulty of optimizing GIOU when the two boxes are far apart and to improve convergence speed. Compared to GIOU, CIOU more accurately considers the position and size offset of the bounding box, especially for small targets and targets with irregular shapes. CIOU demonstrates better robustness and accuracy.

The improved DETR algorithm converges more quickly and enhances DETR’s ability to detect small targets. Data preprocessing is completed based on the characteristics of white blood cell images, and comparative experiments are conducted using different backbone networks. Finally, the performance is compared with that of other algorithms. The results show that the improved method has enhanced the detection accuracy compared to the original algorithm and also exhibits better detection performance and generalization ability compared to other algorithms.

## 3. Methods

**Figure 1:**
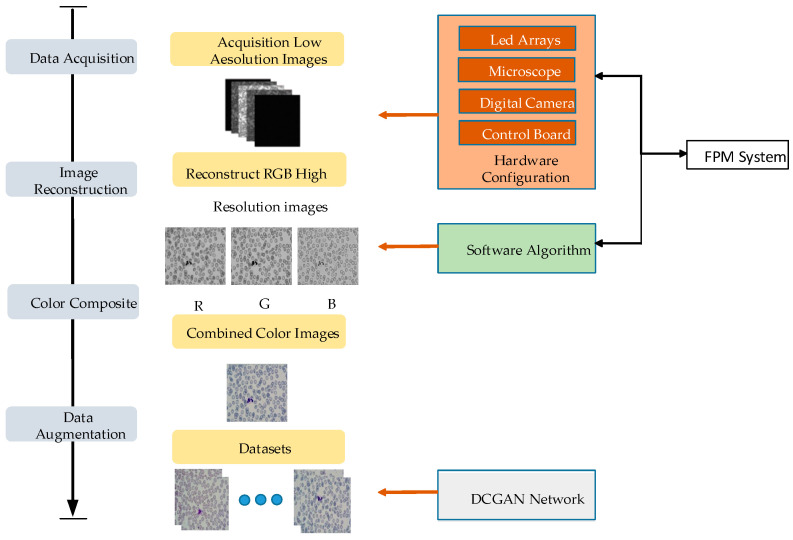
Datasets production of Fourier ptychographic microscopy imaging technology.

**Figure 2:**
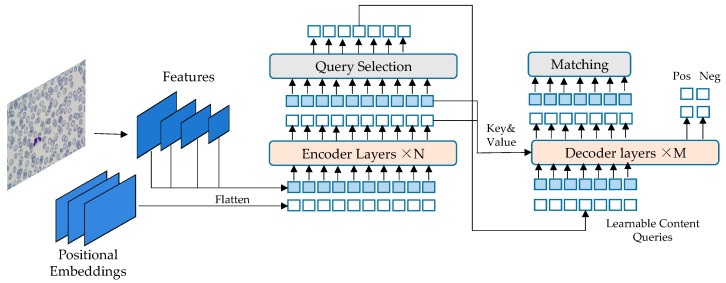
DETR network framework.

In this paper, the FPM system is utilized for collecting high-resolution and wide-field white blood cell images. The collected images are then preprocessed. The improved DETR algorithm is employed for detecting and identifying white blood cells in peripheral blood. To address the problem, a practical neural network classifier is established using the PyTorch framework. The specific process is illustrated in Figure 1 and Figure 2.

The datasets are created using FPM technology, and the programmable LED array illumination module is controlled to replace the light source of the experimental platform. MATLAB software is used to control the LED and provide illumination at different angles. The DMK33UX264 camera is used as an image-acquisition device to capture a large number of low-resolution images that contain white blood cells, which are then saved. The collected RGB three-channel low-resolution images are reconstructed using the FPM algorithm to obtain high-resolution cell images. The peripheral blood cell images are preprocessed to create the basic datasets. The sample datasets are screened to eliminate empty samples and samples without white blood cells. The deep convolution generation adversarial network is used to enhance and label the basic datasets, and the enhanced data is divided proportionally Into a training set and a test set.

The data of peripheral blood leukocytes was collected using FPM. The datasets were obtained through data processing, and then the peripheral blood leukocytes were detected using the improved DETR network. The DETR network structure, shown in Figure 2, consists of two parts: encoder and decoder. The encoder takes as input the image features of blood cells extracted by the CNN, combined with spatial position encoding. These are then passed to the multi-head self-attention module and sent to the feedforward neural network module. Multiple encoder layers can be stacked. The output of the encoder is then sent to the decoder layer through the multi-head mutual attention module between the encoder and decoder. The result is then processed by a feedforward neural network (FNN). Multiple layers of the decoder can also be stacked, and the final output is sent to the FFN for white blood cell prediction and bounding box prediction. The generalization ability of the obtained model is tested using the peripheral blood white blood cell test set.

### 3.1. Fourier Ptychography Microscopic and Reconstruction

Fourier ptychographic microscopy is a novel computational imaging technique that is based on synthetic aperture. This method overcomes the physical limitations and improves the performance of the optical system. It enables coherent imaging with a wide field of view and high resolution. The imaging method mainly utilizes a programmable controlled LED array as the light source to illuminate the samples from various angles. This translation of the sample frequency spectrum in the Fourier domain so that the numerical aperture at the original fixed position obtains a spectrum beyond the numerical aperture of the objective lens. Consequently, the system can collect components that contain high-frequency information about the sample. Sub-aperture overlap is then performed in the frequency domain to calculate the convergence solution of the high-resolution complex amplitude. This method, which replaces spatial mechanical scanning with frequency spectral scanning, not only surpasses the spatial bandwidth product of the objective lens numerical aperture but also enhances the imaging resolution.

**Figure 3:**
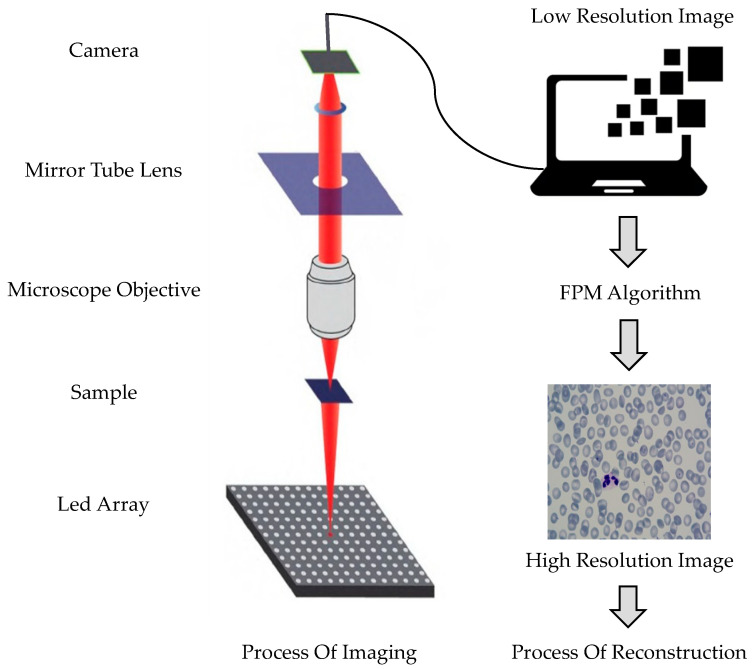
Fourier ptychography microscopic and reconstruction process.

Fourier ptychographic microscopy only requires minor modifications to the traditional microscope. The illumination module of the microscope is replaced with a programmable LED array light source, and a charge-coupled device camera is added. Fourier ptychographic microscopy imaging technology mainly consists of two processes: imaging and reconstruction, as shown in Figure 3. The process involves the LED providing a multi-angle incident light to illuminate the sample, which is then transmitted through the microscope objective and lens barrel. Sub-spectral information is collected at different positions of the frequency spectrum, and the collected information is used to splice the sub-frequency domain, resulting in a high-resolution, wide-field-of-view cell image.

The LED array light source is used for illuminating the sample. It is assumed that the distance between the light source array and the sample is sufficiently far, the light emitting unit on the light source array is small enough, and the emitted light wave is equivalent to a plane wave. When a LED lamp is turned on to illuminate the sample, the wave vector of the incident light is expressed as:

(1) $$k_{n}=\left(\frac{\sin a_{xn}}{\lambda },\frac{\sin a_{yn}}{\lambda }\right),(n=1,2,\ldots ,N_{LED})$$

Among them, (x,y) represents the spatial domain coordinate, $\left(\sin a_{xn},\sin a_{yn}\right)$ represents the incident angle of light, λ represents the wavelength of the incident light, and n=1 represents the normal incidence of LED.

Use o(r) to represent the complex amplitude transmittance function of a single-layer thin sample. When the amplitude of the light source is 1, and the initial phase is 0, the expression for the light source is $\exp(jk_{n}r)$. At this time, the expression for the emergent light after sample modulation is:

(2) $$e(r)=o(r)\exp(jk_{n}r)$$

The spectrum after the Fourier transform is:

(3) $$F\left\{e(r)\right\}=F\left\{o(r)\exp(jk_{n}r)\right\}=O(k-k_{n})$$

F(⋅) represents the Fourier transform, and O represents the sample spectrum distribution. The $O(k-k_{n})$ indicates the movement of the sample spectrum center to $k_{n}$. The position of the LED array light source is inconsistent, resulting in the tilting of the incident light on the sample and causing a shift in the spectrum.

Through the lens coherence transfer function H(k), the spectral distribution of the spectrum in the frequency domain is:

(4) $$G_{n}(k)=O(k-k_{n})H(k)$$

The spectrum is then subjected to an inverse Fourier transform to reach the rear focal plane of the lens, where it is received by the image sensor and converted into a digital signal.

(5) $$I\_n\;(r)={\left|g\_n\;(r)\right|}^{2}={\left|F^{-1}\;(G\_n\;(k))\right|}^{2}$$

The complex amplitude reaching the image plane is denoted as $g_{n}(r)$. Based on the spatial invariance of the coherent imaging system, it can be obtained:

(6) $$I_{n}(r)={\left|g_{n}(r)\right|}^{2}={\left|F^{-1}\left\{G_{n}(k+k_{n})\right\}\right|}^{2}={\left|F^{-1}\left\{O(k)H(k+k_{n})\right\}\right|}^{2}$$

This is equivalent to the translation coherence transfer function rather than the spectrum of the sample. Formula (6) represents the mathematical model of the Fourier stack microscopic imaging system.

### 3.2. DCGAN

Since the peripheral blood cell samples need to be stained, this special production method will result in the target and background in the collected blood cell images not being clearly distinguishable, and the method will cause the blood cells to overlap and distribute densely. These factors can easily interfere with cell detection. Conventional data enhancement methods, such as image cropping, rotation, translation, scaling, contrast change, and noise addition, only increase the number of images and do not significantly enhance the generalization ability of the network model.

DCGAN [44] combines the concept of deep neural networks to optimize the structure of generative adversarial networks (GANs), thereby enhancing the quality and convergence speed of sample generation and producing a wider range of images. In comparison to the GAN model, DCGAN incorporates the concept of a deep neural network, optimizes the network structure, improves the quality of generated samples and improves the network’s convergence speed.

**Figure 4:**
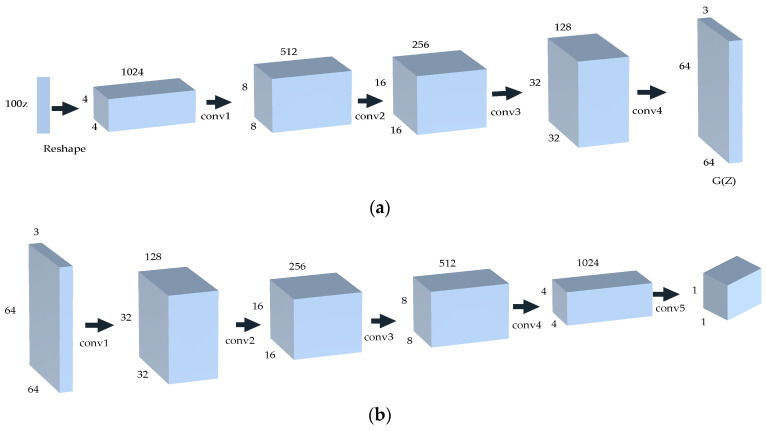
The network structure of DCGAN. (**a**) The generator network; (**b**) the discriminator network.

DCGAN is a direct extension of GAN, which utilizes convolution and convolution transpose layers in the discriminator and generator, respectively. In other words, generator G employs the deconvolution reconstruction technique to recreate the original image during data generation. The discriminator D utilizes convolution technology to identify image features and subsequently make a judgment. The generator receives random noise, which is transformed into a 4 × 4 × 1024 feature map through a fully connected layer. It then passes through four deconvolution layers to produce an image with dimensions of 64 × 64 × 3. The discriminator uses the convolutional layer to downsample the image generated by the generator, resulting in a one-dimensional vector. The network model is depicted in Figure 4.

### 3.3. Network Backbone ResNet50

In the field of target detection, the detected objects exhibit a range of sizes. If simple convolution is employed for image feature extraction, as the number of network layers increases, the number of features associated with small-sized or inconspicuous objects in the deep-level network may be significantly reduced. Consequently, the semantic information is not rich enough, which in turn affects the overall detection accuracy.

The backbone component of the DETR algorithm extracts image features using the ResNet50 residual network. In the ResNet50 network structure, the conv block residual structure utilizes a 1 × 1 convolution kernel and uses an operation with a step size of 2 to achieve feature downsampling. However, this downsampling process using a 1 × 1 convolution kernel leads to information loss. Only certain regions can retain feature information, while features in other regions are unable to participate in the convolution calculation, resulting in the loss of most feature information. For white blood cells in peripheral blood cell images, the lack of abundant feature information makes it difficult for the model to extract relevant information related to the target. As a result, the recognition accuracy of the detection model is reduced.

**Figure 5:**
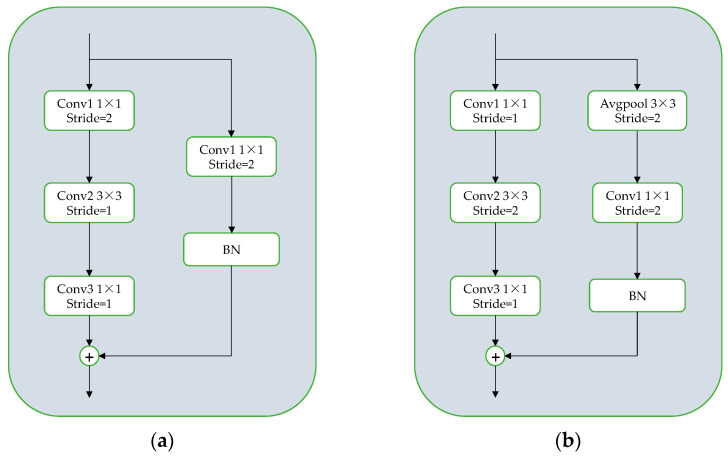
(**a**) The original residual module structure; (**b**) the improved residual module structure.

In order to address the issue of downsampling in the 1 × 1 convolution kernel within the conv block residual structure’s backbone branch, a downsampling process is applied using a 3 × 3 convolution kernel at a stride of 2. Among them, the 1 × 1 convolution has a stride of 1 for feature extraction, while the 3 × 3 convolution kernel is downsampling to minimize feature information loss during the downsampling process. At the residual boundary of the conv block residual structure, an average pooling operation is employed with a stride 2 and a 3 × 3 convolution kernel. Then, a 1 × 1 convolution kernel is added with a stride of 1 for image feature extraction. This ensures that the feature extraction is retained within the average pooling layer and allows for compression of the extracted image features. Since the pooling layer is not controlled by parameters, the potential for overfitting can be effectively reduced, resulting in improved target detection accuracy. The residual structure is illustrated in Figure 5.

### 3.4. Loss Function

The loss of the target detection network includes category loss and border regression loss. The border regression loss function of the DETR network is a combination of the GIOU loss function and the Smooth-L1 loss function. Compared to IOU, GIOU processes the nonoverlapping area of the target, which can fully reflect how the target overlaps and compensates for the deficiency of the IOU boundary loss function in quantifying the real box and the prediction box when they do not intersect. However, when the real target box completely surrounds the prediction box, the relative position relationship cannot be distinguished. At the beginning of training, GIOU needs to enlarge the prediction box and intersect the annotation box, and then begin to reduce the detection result until it coincides with the annotation box. Therefore, it requires an increased number of iterations to converge. CIOU can solve the above problems: The penalty mechanism of CIOU is based on the ratio of the distance between the center point and the diagonal distance. This approach avoids the issue of GIOU being difficult to optimize when the two frames are far apart, resulting in faster convergence speed. Additionally, CIOU can be optimized even when the real target box completely surrounds the prediction box. It takes into account the aspect ratio of both the prediction box and the real target box. The CIOU loss function is as follows:

(7) $$v=\frac{4}{\pi^{2}}(\arctan(\frac{w_{1}}{h_{1}})-\arctan(\frac{w}{h}){)}^{2}$$

(8) $$a=\frac{v}{(1-IOU)+v}$$

(9) $$L_{ciou}=1-IOU+\frac{D^{2}}{C^{2}}+av$$

Among them, $w_{1}$ and $h_{1}$ represent the width and height of the real box, while w and h represent the width and height of the detected box, D refers to the Euclidean distance between the center point of the detected box and the target box, C represents the diagonal length of the minimum circumscribed moment of the detected box and the target box. Therefore, the improved DETR border loss function can be expressed as the following formula:

(10) $$L_{box}(b_{i},{\hat{b}}_{s(i)})=\lambda_{CIOU}L_{CIOU}(b_{i},{\hat{b}}_{s(i)})+\lambda_{ll}L_{l1}(b_{i},{\hat{b}}_{s(i)})$$

Among them, $b_{i}$ and ${\hat{b}}_{s(i)}$ represent the real box coordinates of the i target and the s(i) detection box coordinates predicted by the algorithm, respectively.

## 4. Experiments and Results

### 4.1. Image Acquisition and Reconstruction

**Figure 6:**
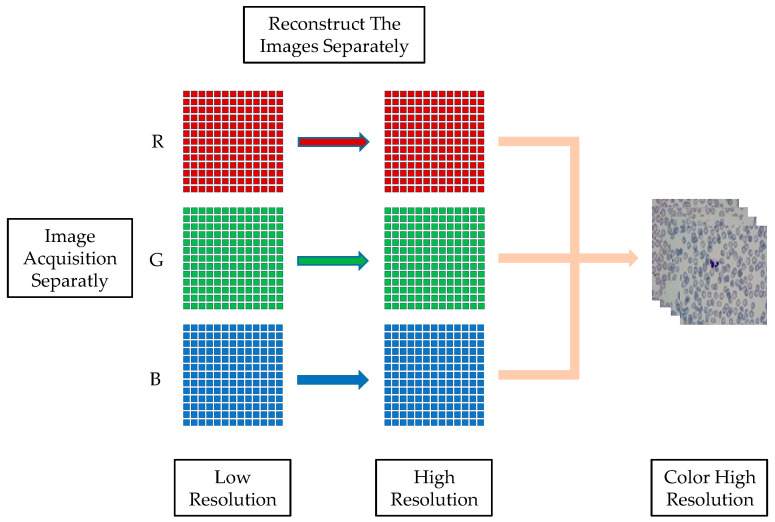
FPM color image reconstruction.

The key to detecting peripheral blood cells is understanding how to use the FPM system to collect low-resolution images. In the FPM system, the blood smear sample is placed in the central position of the stage. The brightness is adjusted by lighting the LED array light source, and the focus is adjusted to obtain the clearest image. To integrate the integration of the Fourier stack microscopic imaging system, the upper computer program and reconstruction algorithm of the LED array light source are integrated using MATLAB software to complete the image acquisition and reconstruction. The running program controls the LED to light up for low-resolution image acquisition and saves the acquired image to the corresponding folder for the reconstruction algorithm. Each LED illuminates the sample with three color light sources (red, green, and blue) to obtain a color image. Therefore, a total of 13 × 13 × 3 (507) low-resolution images under three colors, red, green, and blue, will be collected. After applying the spectrum reconstruction algorithm, the high-resolution grayscale images for each of the RGB colors are reconstructed, respectively. Finally, the reconstruction results are synthesized to create high-resolution color images. The process is illustrated in Figure 6.

### 4.2. Data Preprocessing

The training datasets required to train the neural network model are very large, and the required sample size must be large enough. The bigger the number of samples, the better the effect of the trained model and the stronger the generalization ability of the model. The Fourier ptychographic microscopic imaging system needs to collect 507 low-resolution images each time and then reconstruct them in order to obtain a color high-resolution cell image, which makes the process very time-consuming. Because the peripheral blood cell image is taken under the microscope, this uniqueness results in obvious differences in the target characteristics of the image data compared to the commonly used target detection datasets VOC (visual object class) and COCO (common objects in context).

**Figure 7:**
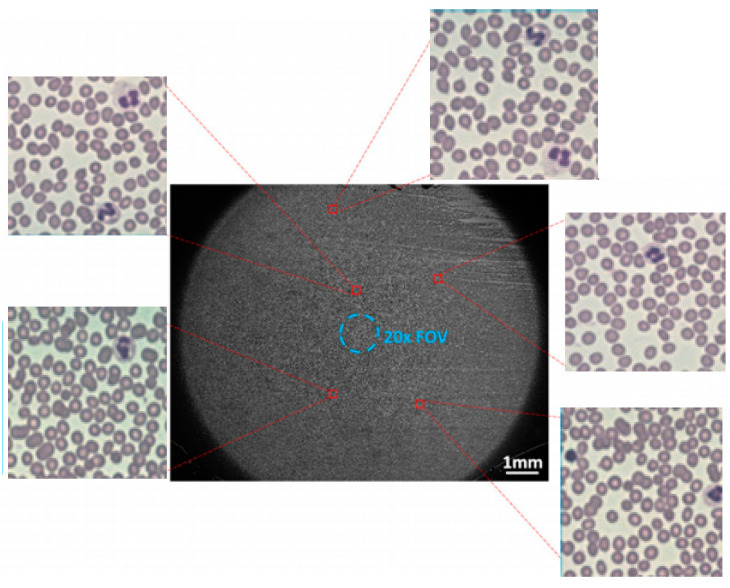
FPM peripheral blood cell image.

As depicted in Figure 7, this is a high-resolution, wide-field peripheral blood cell image obtained through FPM. The figure reveals that there is not a clear distinction in features between the target to be detected in the cell image and the background of the image. Additionally, the image contains overlapping cells. The background is intricate, and the target to be detected is of small size, with a dense distribution of small targets. These factors can potentially disrupt cell detection.

**Figure 8:**
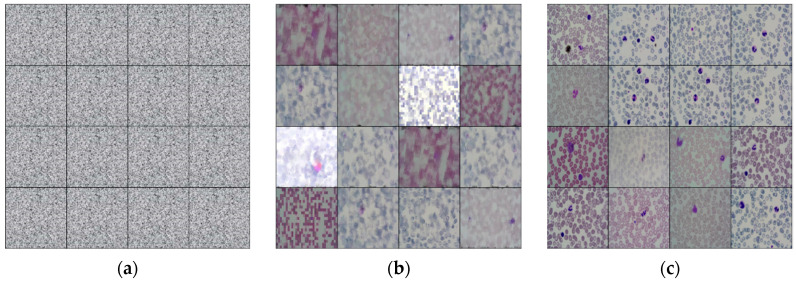
White blood cell enhancement example diagram of DCGAN network. (**a**) The initial training result; (**b**) the image with obvious contour features obtained by the model after 170 rounds of training; (**c**) the resulting image after training.

To enhance the quality and quantity of peripheral blood white blood cell image datasets, the experimental objective is to employ the DCGAN network for generating a specific number of white blood cell images. These images will aid the target detection network in identifying white blood cells. To avoid potential issues with overfitting due to excessive generated data, the collected white blood cell datasets are expanded in a 1:1 ratio. This experiment is designed to have 300 training iterations, and images with distinct contour features can be generated by the 170th iteration. The corresponding iterative effect is depicted in Figure 8.

### 4.3. Experimental Result

#### 4.3.1. Comparison of Data before and after Preprocessing

**Figure 9:**
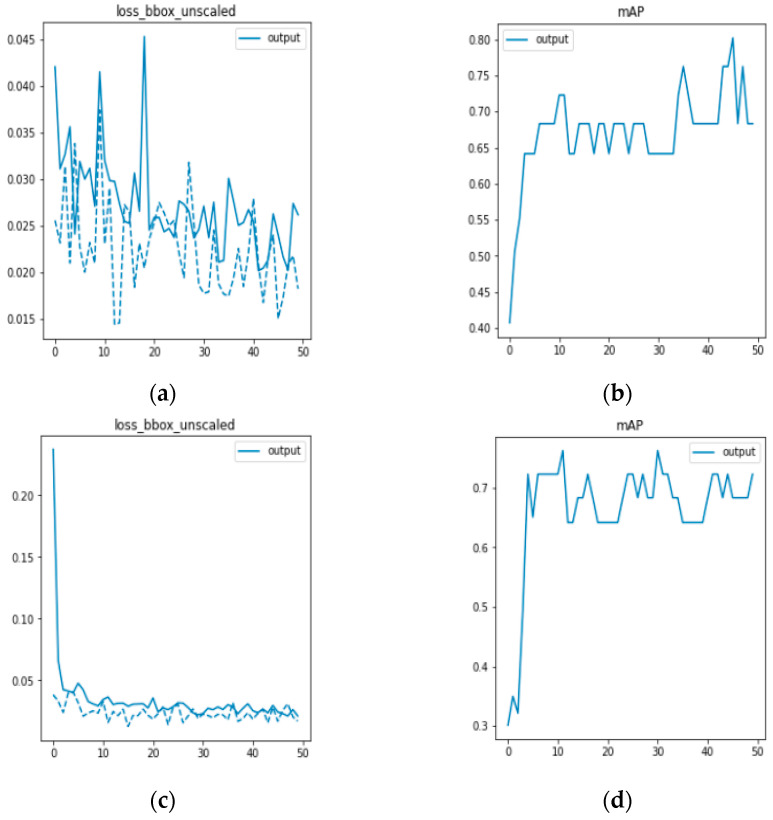
(**a**,**b**) are DETR model training; (**c**,**d**) are DETR model training after data preprocessing.

In this experiment, the effectiveness of the DETR network model and the generalization ability of the generated data to the network model are verified. The datasets used are the data collected by the Fourier laminated microscopic imaging system and the data preprocessed by the DCGAN network. Both datasets are input into the DETR network for comparison, as shown in Figure 9. Figure 9a,b represent the bounding loss function and mAP of the training results of the DETR model, while Figure 9c,d represent the training results of the DETR model after data preprocessing.

Because the blood cell image has not been preprocessed, there are errors between the prediction frame and the calibration frame due to cell overlap, adhesion, and complex background, resulting in a large fluctuation. After preprocessing the data, the frame loss function rapidly converges and steadily decreases, leading to more accurate detection of white blood cells. Figure 9d shows that after data preprocessing, there is less fluctuation and a steady increase in mAP, indicating improved accuracy in detecting white blood cell targets. In this section, the effectiveness of the experiment was verified by training and comparing the results of 50 epochs. The comparison data are presented in Table 1.

It can be observed from Table 1 that the DETR network model is capable of detecting peripheral blood leukocytes. The mean average precision (mAP) value of the DETR network is 0.746 when only the data collected by the Fourier ptychographic imaging system are used for training. After DCGAN network data enhancement, the mAP value of the model is 0.794, representing an increase of 4.8 percentage points. The experimental results demonstrate that the DETR network is effective in detecting human peripheral blood leukocytes. Based on the DCGAN network, a clearer image of peripheral blood cells can be generated, thereby significantly enhancing the generalization ability of the trained model and improving the accuracy of white blood cell detection.

#### 4.3.2. Comparison of Different Backbone Networks

**Figure 10:**
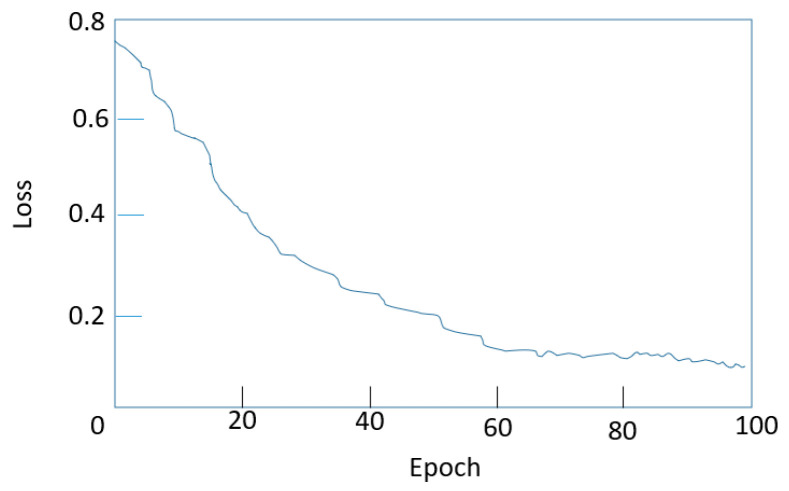
DETR model training process of the loss function.

In the process of this experiment, considering that the DETR algorithm first extracts the network through the CNN feature and then inputs the feature map and position coding into the encoder and decoder together. Therefore, the different feature extraction network models were used for comparison. As shown in Figure 10, it is the loss function curve in the training process of the DETR model. From the graph, it can be seen that before the number of iterations reached 70 epochs, the loss value of the network had been in a stable and rapid decline state. When the number of iterations reached 70, the loss value had been fluctuating in a small range and basically no longer changed. At this time, the network had begun to converge, the network training process was normal, and the model performance had reached its best.

Different feature extraction networks are selected and deepened from ResNet18 to ResNet101. The experimental results are shown in Table 2. In terms of accuracy, ResNet101 had the highest mAP value, followed by ResNet50. In terms of parameters, ResNet18 has the smallest number of parameters but the lowest accuracy, while ResNet101 has the largest number of parameters and the highest accuracy. The network’s depth affects its representation ability, with deeper networks generally having stronger feature expression abilities. However, deeper models are more complex and require longer training times. Therefore, it is necessary to select a reasonable network depth to minimize the training time while ensuring the performance of the model. ResNet101 has high accuracy as a feature extraction network, but it has the largest network model. The ResNet18 network model is small but has the lowest accuracy. In comparison, ResNet50 is selected as the feature extraction network of the DETR network. It yields superior results, attains can achieve higher average accuracy, and the parameters are within an acceptable range.

#### 4.3.3. Ablation Experiment

After adding different improvement strategies to the DETR algorithm, the average accuracy of training, parameters, and the number of frames per second (FPS) are shown in Table 3. The mAP of the DETR algorithm is 0.794 without any improvement measures. To obtain more cell image feature information, the residual module of the backbone was improved, increasing the mAP value by 5.4 percentage points. To avoid the problem of GIOU being difficult to optimize when the two boxes are far away, CIOU was introduced as the border loss function, increasing the mAP value by 9.1 percentage points. After synthesizing the above two improvement points, the improved DETR model proposed in this paper effectively realizes the achieved mAP value of 0.936, demonstrating the practicability and effectiveness of the improved method for the DETR model. It can be seen from the results that the improved DETR model is superior to the original DETR model in terms of mAP, parameter quantity, and FPS. Therefore, it is utilized in the detection of white blood cell detection greater accuracy, a smaller model, and faster reasoning speed.

#### 4.3.4. Comparison of Different Network Model Training

To demonstrate the effectiveness of the proposed improved DETR algorithm, it is compared with the mainstream network model in the field of computer vision in terms of performance. This comparison aims to showcase the improvement of the method proposed in this paper for white blood cell detection, as well as the advantages and disadvantages of the improved method compared with to models. Table 4 presents a comparison between the trained improved DETR model and the current traditional target detection algorithm. The improved DETR algorithm achieves an mAP of 0.936, has a model parameter of 92 M, and operates at a FPS of 58. After training, the improved architecture exhibits the highest average accuracy, the smallest model size, and the fastest inference speed. It outperforms SSD, YOLOv5, and other methods, enabling more accurate detection of white blood cells in peripheral blood.

**Figure 11:**
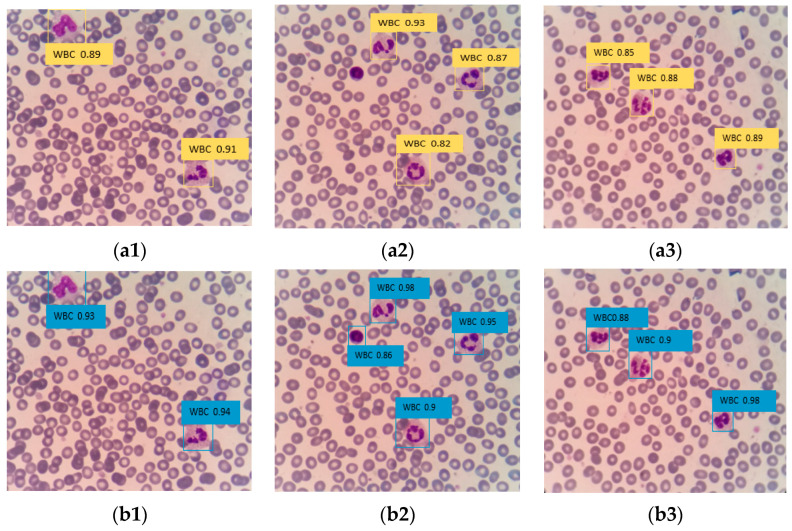
(**a1**–**a3**) is Faster R-CNN model training; (**b1**–**b3**) is improved DETR model training.

The Faster R-CNN algorithm, which has higher detection accuracy, was selected to compare its detection effect with the improved DETR algorithm. The images were inputted into the architecture using different training models to obtain the detection and recognition results, as shown in Figure 11. In Figure 11(a2), a white blood cell was missed in the detection by Faster R-CNN, whereas in Figure 11(b2), there was no missed detection in the improved DETR. Additionally, the confidence of the improved DETR in detecting white blood cells was higher than that of the Faster R-CNN algorithm. This shows that the application of the improving DETR algorithm in the medical field has a good effect and significantly improves the detection accuracy and speed of peripheral blood white blood cells.

## 5. Conclusions

In this paper, peripheral blood leukocyte detection based on an improved DETR algorithm is proposed. The FPM can compensate for the limitations of traditional microscope resolution and field of view. It only requires a simple transformation of the traditional microscope to synthesize a high-resolution, wide-field white blood cell image in a low-resolution image obtained without mechanical scanning.

The DCGAN network preprocesses the peripheral blood cell data, improving the quality of the cell image dataset and facilitating detection. The experimental results demonstrate that the mAP value reaches 0.746 after training and testing using the DCGAN network for data enhancement.

In the ResNet50 backbone network, the residual structure of the backbone branch has been modified, and the average pooling operation is adopted to retain the feature information of the small cell target. CIOU addresses the issue of GIOU being difficult to optimize when the two boxes are far away and the convergence speed is faster. The final mAP value has increased by 14.2 percentage points. The ablation experiment has confirmed the effectiveness of the improved DETR residual structure and loss function in the model. Additionally, when compared to the existing target detection networks, the algorithm also surpasses the classical CNN detection algorithm in terms of parameters, detection accuracy, and FPS. It achieves high-precision detection of peripheral white blood cells.

The model introduces the excellent DETR in machine vision into the field of medical images. The improved DETR demonstrates superior detection performance for small targets, thus confirming its viability in microscopic medical image detection. Considering the accuracy and detection performance of the proposed method, it can be concluded that it has the potential to simplify the artificial blood cell recognition process. This method offers assurance for future biomedical research, including cell counting and classification. It is a useful attempt to introduce it into the field of medical images.

Although we have achieved excellent performance in experimental comparisons with other detection models, there is a minor issue in the cell image where a few white blood cells are not fully exposed at the edge. This leads to missed detections, but it does not impact the overall results. In order to meet the high standards of medicine, we are working on improving our network structure to achieve flawless detection results. In the future, clinical data for specific diseases (such as leukemia) will be sought, and more blood cell datasets will be collected for verification to expand the applicability of the model.

## Figures and Tables

**Table 1:** Comparison of model detection before and after experiment and treatment.

Model	mAP
DETR	0.746
DETR + DCGAN	0.794

**Table 2:** The results of different backbone networks.

Backbone	mAP	Size/M
DETR (ResNet18)	0.685	57.2
DETR (ResNet34)	0.743	63.5
DETR (ResNet50)	0.794	77.3
DETR (ResNet101)	0.817	85.8

**Table 3:** The results of different improvement strategies.

Improvement	mAP	Size/M	FPS
DETR + Residuals	0.848	113	45
DETR + CIOU	0.885	106	56
DETR + Residuals + CIOU	0.936	92	58

**Table 4:** The results of different network model training.

Improvement	mAP	Size/M	FPS
SSD	0.823	100	28
YOLOv5	0.834	245	40
YOLOv6	0.865	140	55
Faster R-CNN	0.882	108	18
DETR + Residuals + CIOU	0.936	92	58

## Data Availability

Not applicable.
